# Quercetin Protects Intestinal Barrier Integrity in Inflammation and Oxidative Stress

**DOI:** 10.3390/nu18132169

**Published:** 2026-07-03

**Authors:** Olugbenga Balogun, Hye Won Kang

**Affiliations:** Food and Nutritional Sciences, Department of Family and Consumer Sciences, North Carolina Agricultural and Technical State University, Greensboro, NC 27411, USA; balogun340@gmail.com

**Keywords:** quercetin, intestine, inflammation, tight junction

## Abstract

**Background/Objective:** An obesogenic diet triggers intestinal inflammation and oxidative stress, leading to epithelial barrier dysfunction and increased risk of metabolic disorders. This study investigated the mechanisms by which quercetin protects intestinal integrity in high-fat diet (HFD)–fed mice. **Methods:** Mice were fed an HFD or a low-fat diet (LFD) with or without 1% quercetin, intestinal gene and protein expression, microRNA levels, permeability, and circulating intestinal biomarkers were assessed. **Results:** Mice fed an HFD with quercetin (HFDQ) showed a 17% improvement in intestinal barrier integrity with increased expression of tight junction and mucin genes and proteins. The nuclear translocation of the nuclear factor-κB (NF-κB) p65 subunit in the ileum decreased by 34%, whereas its acetylation was reduced by 50–57% throughout the intestine, with downregulation of NF-κB-regulated pro-inflammatory genes and proteins. Quercetin increased the nuclear factor erythroid 2-related factor 2 (NRF2) by ~ 25% across intestinal segments and upregulated antioxidant enzyme genes. It suppressed toll-like receptor 4 (TLR4) by 50% and restored AMP-activated protein kinase (AMPK) and sirtuin 1 to levels comparable to those in LFD mice. Altered microRNAs (miRNA-16, 200b, 122, 34a, and 21) supported these molecular changes. Quercetin also restored short-chain fatty acid receptors and serotonin transporters that were affected by HFD. Plasma lipopolysaccharide (LPS), cluster of differentiation 14, LPS-binding protein, and myeloperoxidase activity decreased by 36, 31, 42, and 37%, while glucagon-like peptide-1 increased by 23%. **Conclusions:** Quercetin protects epithelial barrier integrity against HFD-induced intestinal inflammation and oxidative stress via the AMPK-mediated NF-κB and NRF2 signaling pathways.

## 1. Introduction

The intestinal epithelium serves as a critical interface between the host and the luminal environment by permitting the absorption of nutrients while restricting the passage of harmful substances, including proinflammatory compounds such as lipopolysaccharide (LPS) [1,2]. Maintenance of epithelial barrier integrity depends largely on tight junctions (TJs), which are multiprotein complexes located at the apical membrane between neighboring epithelial cells. TJs consist of transmembrane proteins, including occludin (OCLN) and claudins (CLDNs), linked to scaffold proteins such as zonula occludens 1 (ZO1), which together control paracellular permeability and prevent the passage of luminal microorganisms and toxins into underlying tissues [3]. Therefore, interventions that attenuate intestinal inflammation and preserve tight junction integrity may help maintain epithelial barrier function.

Dietary polyphenols exert protective effects on intestinal barrier function through antioxidant and anti-inflammatory properties [4]. Quercetin, commonly found in various fruits and vegetables but also abundant in onion peel, a byproduct of onion exhibited strong antioxidant and anti-inflammatory effects. Quercetin alleviated LPS—or chemically induced intestinal inflammation by activating nuclear factor erythroid 2-related factor 2 (NRF2)/heme oxygenase 1 (HO1) signaling and suppressing nuclear factor-κB (NF-κB)-mediated cytokine expression [5,6]. These effects are associated with the restoration of intestinal morphology and increased expression of TJ proteins such as ZO1, OCLN, and CLDN1 [5,6]. Previously, we also reported that quercetin and onion peel extract prevented LPS-induced TJ disruption by increasing *ZO1*, *OCLN*, *CLDN1*, and *CLDN4* expression in Caco-2/HT-29 cells through the activation of AMP-activated protein kinase (AMPK) and inhibition of NF-κB via the AMPK–silent mating type information regulation 2 homolog 1 (SIRT1) signaling pathway [7]. However, the roles of microRNAs (miRNAs), gut-derived hormones, and microbial metabolites in quercetin-mediated intestinal protection remain incompletely understood. Gut hormones, including glucagon-like peptide 1 (GLP1), GLP2, peptide YY, and serotonin (5-hydroxytryptamine, 5-HT), are essential for epithelial repair, barrier maintenance, and mucosal immune defense. Quercetin improved gut microbiota composition and modulated microbial metabolites such as short-chain fatty acids (SCFAs) in diet-induced obese mice [8]. Therefore, the present study aims to elucidate the mechanisms by which quercetin preserves epithelial barrier function by attenuating intestinal inflammation and oxidative stress *in vivo*, with a particular focus on miRNA regulation and the assessment of gut-derived hormonal and metabolite receptors to explore their potential associations with barrier function improvement.

## 2. Materials and Methods

### 2.1. Animals and Diets

Four-week-old male C57BL/6 mice were obtained from Charles River Laboratories (Durham, NC, USA). Quercetin (Purity is ≥95%, cat# Q4951) was purchased from Sigma-Aldrich (St. Louis, MO, USA). The quercetin-supplemented diets were prepared by thoroughly mixing quercetin powder into a low-fat diet (LFD, 10% kcals from fat) and a high-fat diet (HFD, 60% kcals from fat) at a concentration of 1% by weight (10 g quercetin per kg diet) (TestDiet, Richmond, IN, USA). After one week of acclimation (21 ± 1 °C, 55% ± 5% humidity, and a 12 h light/dark cycle), mice were divided into four groups and fed a LFD (*n* = 9), LFD supplemented with 1% quercetin (LFDQ) (*n* = 9), HFD (*n* = 9), and HFD supplemented with 1% quercetin (HFDQ) (*n* = 9) [8]. The diet was provided to mice in solid-pellet form ad libitum throughout the study. Body weight and food consumption were monitored weekly throughout the 12-week intervention. At the end of the study, mice were fasted for 16 h and euthanized by CO_2_ inhalation. Blood was subsequently collected by cardiac puncture using syringes containing 250 mM EDTA. Following the collection, the samples were centrifuged at 12,000× *g* for 10 min at 4 °C to separate the plasma. The plasma was then stored at −80 °C for further use. The duodenum (DUE), jejunum (JEJU), and ileum (ILE) of the intestine were separated, snap-frozen, and stored in liquid nitrogen. Animal handling and anesthesia protocols were approved by the Institutional Animal Care and Use Committee of North Carolina A&T State University (LA20-005).

### 2.2. Intestinal Permeability Assay

Intestinal permeability was assessed using a fluorescein isothiocyanate (FITC)-dextran [9]. Mice were deprived of food for 6 h and orally gavaged with (FITC)-dextran (4 kDa, Sigma-Aldrich) dissolved in saline at a dose of 500 mg/kg body weight. After 4 h, blood samples were collected via submandibular bleeding and centrifuged at 1200× *g* for 3 min at 4 °C to obtain plasma. Plasma samples were diluted with PBS (1:3 *v*/*v*), and the FITC-dextran fluorescence was quantified using a SpectraMax M3 microplate reader (Molecular Devices, San Jose, CA, USA) with excitation and emission wavelengths at 485 nm and 535 nm, respectively.

### 2.3. Biochemical Analysis

The plasma intestinal fatty-acid-binding protein (I-FABP, MBS035456), LPS (MBS261094), peptide YY (MBS725360), LPS-binding protein (LBP, ab213876), cluster of differentiation 14 (CD14, ab242236), myeloperoxidase (MPO, ab105136), and GLP1 (RAB0201) concentrations were determined using the enzyme-linked immunosorbent assay (ELISA) according to the manufacturer’s instructions (MyBioSource, Inc., San Diego, CA, USA, Abcam, Waltham, MA, USA, and Sigma-Aldrich). Briefly, standards and plasma samples (*n* = 9 per diet group) were added to the antibody-coated microplate, followed by a horseradish peroxidase (HRP)-conjugated detection reagent, and the plate was incubated at 37 °C for the time specified by each kit. After washing, chromogenic substrates were added, and the plate was incubated at 37 °C for the time required by each kit. The reaction was terminated with stop solution, and the absorbance was measured at 415 nm for MPO and at 450 nm for I-FABP, LPS, LBP, CD14, and GLP1. To minimize potential matrix-related interference, all ELISAs were performed according to validated manufacturer protocols and under standardized conditions.

### 2.4. Quantitative Polymerase Chain Reaction (qPCR) Analysis

Total RNA was isolated from each intestinal segment using TRIzol reagent (Thermo Fisher Scientific, Waltham, MA, USA) according to the manufacturer’s instructions [7]. To synthesize cDNA, total RNA (1 µg) from each sample was mixed with oligo (dT) and water, and then subjected to reverse transcription using the GoScript reverse transcription system (Promega, Madison, WI, USA) in a SimpliAmp^TM^ Thermal Cycler (Thermo Fisher Scientific) [7]. A cDNA mix was added to the RNA solution, followed by cycling conditions of 25 °C for 5 min, 42 °C for 60 min, and 70 °C for 15 min. Gene expression was assessed using the FastStart Essential DNA SYBR Green Master Mix Kit, with a master mixture prepared by combining primers and SYBR Green master mix (Roche Diagnostics, Indianapolis, IN, USA), which was then added to a 96-well PCR plate along with cDNA in each well. The PCR amplification was conducted with denaturation at 95 °C for 10 s, annealing at 60 °C for 10 s, and extension at 72 °C for 10 s, for 40 cycles, in a Light Cycler 96 (Roche Diagnostics) [7]. Ribosomal protein L32 (*Rpl32*) was used as a housekeeping gene.

### 2.5. miRNA Analysis

The miRNAs directly targeting toll-like receptor 4 (TLR4), inhibitor of nuclear factor kappa-B kinase subunit beta (IKKB), and tight junctions were identified using target prediction tools (TargetScan and MiRDB) [10]. Total RNA was extracted, and cDNA was synthesized using miRCURY LNA RT kit (Qiagen, Valencia, CA, USA). Briefly, 10 ng of total RNA was combined with reverse transcriptase and 5× reaction buffer. The mixture was incubated at 42 °C for 60 min, followed by 95 °C for 5 min, and then cooled to 4 °C. miRNA expression levels were determined by qPCR using miRCURY LNA SYBR Green PCR kit (Qiagen). Briefly, 3 μL of cDNA (diluted 1:30) was mixed with 5 μL of SYBR PCR Master Mix, 1 μL of PCR primer mix, and 1 μL of water to achieve a total volume of 10 μL. The reaction mixture was incubated at 95 °C for 2 min, followed by 45 cycles of 95 °C for 10 s and 56 °C for 60 s in a Light Cycler 96 (Roche Diagnostics). U6 small nuclear RNA (snRNA) (Qiagen) was used as a reference gene.

### 2.6. Western Blot Analysis

Total proteins were extracted from each segment of the intestine using RIPA lysis buffer. To determine NF-κB activation, cytoplasm and nuclear were isolated using a NE-PER Nuclear and Cytoplasmic Extraction Reagent Kit (Thermo Fisher Scientific, Waltham, MA, USA) according to the manufacturer’s instructions. Western blot analysis was performed as previously described [7]. Primary antibodies, pAMPK, AMPK, ZO1, CLDN1, SIRT1, NF-κB p65, acetyl p65 (1:1000; Cell Signaling, Danvers, MA, USA) and β-ACTIN (1:3000; Sigma-Aldrich) were used. The HRP-conjugated secondary antibodies, sheep anti-rabbit IgG (1:5000) for pAMPK, AMPK, ZO1, CLDN1, SIRT1, NF-κB p65, acetyl p65 (Novus Biology, Littleton, CO, USA) and goat anti-mouse IgG (1:5000) for β-ACTIN (Thermo Fisher Scientific) were used. The target proteins were detected using enhanced chemiluminescence (Thermo Fisher Scientific) using the ChemiDoc XRS System (Bio-Rad, Hercules, CA, USA). The density of each band was analyzed using the National Institutes of Health ImageJ program (Version 1.54).

### 2.7. Statistical Analysis

The data were presented as mean ± standard deviation (SD). All experiments were performed in triplicate. Prior to statistical analysis, normality of the data distribution was assessed using the Shapiro–Wilk test, and homogeneity of variance was evaluated using Brown–Forsythe test (Prism 11.02, GraphPad Software, La Jolla, CA, USA). One-way ANOVA with Tukey’s post hoc test (Prism 11.02, GraphPad Software) was used to analyze the difference between groups. *p* < 0.05 was considered significant.

## 3. Results

### 3.1. Effect of Quercetin on Inflammatory Biomarkers and Intestinal Hormones

**Figure 1:**
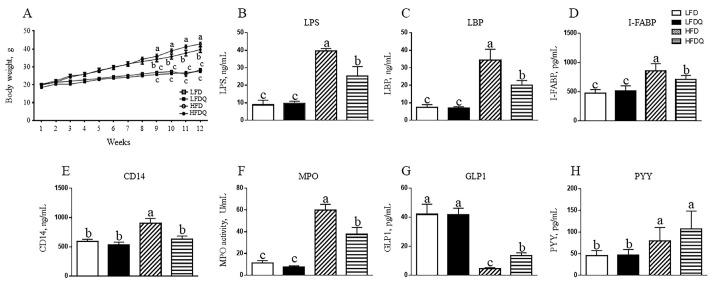
Effect of quercetin on body weight, inflammatory biomarkers and intestinal hormones. (**A**) Body weight (One-way ANOVA followed by Tukey’s post hoc test was performed separately at each time point to compare differences among the four experimental groups), Plasma (**B**) LPS, (**C**) LBP, (**D**) I-FABP, (**E**) CD14, (**F**) MPO activity, (**G**) GLP1, and (**H**) PYY. Data are presented as mean ± SD (*n* = 9/group). Groups not sharing a common letter are significantly different according to Tukey’s post hoc test (*p* < 0.05).

HFD and HFDQ-fed mice did not show any body weight difference up to week 8, but there was a significant decrease in the body weight of HFDQ-fed mice compared to HFD-fed mice from week 9 to week 12 (*p* < 0.05). At week 12, HFDQ-fed mice showed a 10% decrease in body weight as compared to HFD-fed mice (Figure 1A). There was no difference in the body weight of LFD- and LFDQ-fed mice. HFD-induced increases in plasma LPS, LBP, and I-FABP levels, as well as serum CD14 levels, were decreased by 36% (*p* < 0.0001), 42% (*p* = 0.028), 17% (*p* = 0.007), and 31% (*p* = 0.013), respectively, with quercetin supplementation (Figure 1B–E). Plasma MPO activity was reduced by 37% (*p* = 0.005) in HFDQ-fed mice, compared to HFD-fed mice (Figure 1F). In HFDQ-fed mice, plasma GLP1 levels reduced by HFD were restored by 21%, and, interestingly, PYY levels were not changed (Figure 1G,H)

### 3.2. Effect of Quercetin on Genes Related to Inflammation and Cellular Antioxidants in the Intestine

**Figure 2:**
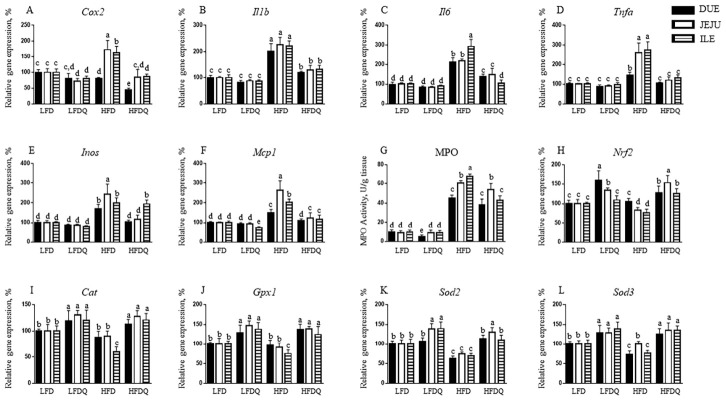
Effect of quercetin on genes related to inflammation and cellular antioxidant in the intestine. (**A**) *Cox2*, (**B**) *Il1b*, (**C**) *Il6*, (**D**) *Tnfa*, (**E**) *Inos*, (**F**) *Mcp1*, (**G**) MPO activity, (**H**) *Nrf2*, (**I**) *Cat*, (**J**) *Gpx1*, (**K**) *Sod2*, and (**L**) *Sod3*. Data are presented as mean ± SD (*n* = 9/group). Groups not sharing a common letter are significantly different according to Tukey’s post hoc test (*p* < 0.05).

To determine the effect of quercetin on intestinal inflammation, the expression of pro-inflammatory genes was measured (Figure 2A–F). In LFD-fed mice, quercetin supplementation decreased cyclooxygenase-2 (*Cox2*) expression by 20-25% in the JEJU and ILE compared to LFD-fed controls (Figure 2A). HFD feeding increased *Cox2* expression by 73% in the JEJU (*p* = 0.003) and 64% in the ILE (*p* = 0.02), but these increases were prevented by quercetin supplementation. HFD feeding caused significant increases in interleukin 1 beta (*Il1b*), *Il6*, tumor necrosis factor alpha (*Tnfa*), and inducible nitric oxide synthase (*Inos*) expression in the intestine compared to LFD-fed mice (Figure 2B–E). Quercetin supplementation prevented HFD-induced *Il1b* and *Il6* expression by 69-100% across all intestinal segments (Figure 2B,C). Moreover, *Tnfa* expression in the intestine of HFDQ-fed mice exhibited the same expression levels as LFD-fed mice (Figure 2D). Although quercetin did not change *Inos* expression in the ILE, *Inos* expression was decreased by 40% and 52% in the DUE and JEJU (Figure 2E). As shown in Figure 2F, HFD feeding increased *Mcp1* expression by 51%, with even greater induction in the JEJU and ILE. This rise was fully restored to control levels in DUE and reduced by 53% and 33% in the JEJU and ILE. HFD-fed mice showed a significant increase in MPO activity by approximately 368-596% across intestinal segments, whereas quercetin supplementation reduced MPO activity by 42% (*p* = 0.0002), particularly in the ILE (Figure 2G). To determine the effect of quercetin on HFD-induced oxidative stress in the intestine, the expression of genes involved in redox regulation was measured (Figure 2H–L). *Nrf2*, a transcription factor of the primary pathway to regulate cells’ redox state, was elevated by 60% in the DUE and 34% in the JEJU of LFDQ-fed mice compared to LFD-fed mice (Figure 2H). HFD feeding resulted in a 17-23% reduction in *Nrf2* expression in the JEJU and ILE. However, HFDQ-fed mice exhibited greater *Nrf2* expression across all intestinal segments than LFD-fed mice (Figure 2H). Quercetin supplementation in LFD-fed mice upregulated the intestinal expression of catalase (*Cat*), glutathione peroxidase 1 (*Gpx1*), superoxide dismutase 2 (*Sod2*), and *Sod3* genes, which encode key antioxidant enzymes (Figure 2I–L). *Cat* and *Gpx1* expression were reduced in the ILE of HFD-fed mice, whereas *Sod2* and *Sod3* genes were downregulated across all intestinal segments and in the DUE and ILE, respectively. These reductions in HFD-fed mice were fully restored to levels similar to or greater than those in LFD-fed mice (Figure 2I–L).

### 3.3. Effect of Quercetin on Intestinal Integrity, Short-Chain Fatty Acid Receptors, and Serotonin Transporters

**Figure 3:**
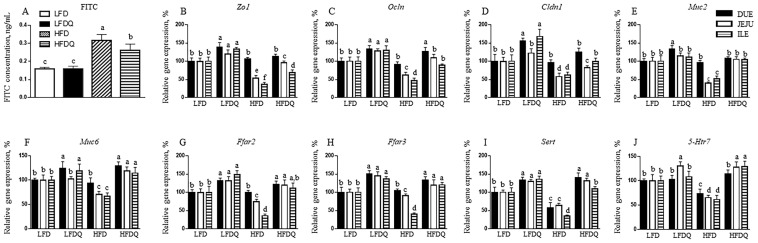
Effect of quercetin on genes related to tight junction, short-chain fatty acid receptors, and serotonin in the intestine. (**A**) FITC, (**B**) *Zo1*, (**C**) *Ocln*, (**D**) *Cldn1*, (**E**) *Muc2*, (**F**) *Muc6*, (**G**) *Ffar2*, (**H**) *Ffar3*, (**I**) *Sert*, and (**J**) *5-Htr7*. Data are presented as mean ± SD (*n* = 9/group). Groups not sharing a common letter are significantly different according to Tukey’s post hoc test (*p* < 0.05).

To determine the effect of quercetin on intestinal integrity, intestinal permeability and genes related to tight junction and mucin production were examined. HFDQ-fed mice exhibited a 17% decrease in FITC permeability compared to HFD-fed mice (Figure 3A). In LFD-fed mice, quercetin supplementation upregulated tight junction genes, *Zo1*, *Ocln*, and *Cldn1* across all intestinal segments (Figure 3B–D). HFD feeding reduced the expression of *Zo1*, *Ocln*, and *Cldn1* in the JEJU and ILE, but this reduction was prevented by quercetin supplementation. LFDQ-fed mice showed an increased *Muc2* gene in the DUE by 33%, while there was no change in the JEJU and ILE as compared with LFD-fed mice (Figure 3E). Quercetin increased *Muc6* gene by 24% in the DUE and 18% in the ILE of LFD-fed mice (Figure 3F). HFD feeding decreased *Muc2* and *Muc6* expression in the JEJU and ILE, but quercetin protected against these reductions (HFD JEJU vs. HFDQ JEJU, *p* < 0.0001; HFD ILE vs. HFDQ ILE, *p* = 0.0028 in *Muc2*). Notably, *Muc6* expression in the intestine of HFDQ-fed mice was higher than in LFD-fed mice (Figure 3F). To determine whether quercetin regulates intestinal integrity via microbial metabolites, the expression of free fatty acid receptor 2 and 3 (*Ffar2* and *Ffar3*) genes, which encode short-chain fatty acid receptors, was measured. Compared to LFD-fed mice, LFDQ-fed mice showed upregulated *Ffar2* and *Ffar3* genes (Figure 3G,H). HFD feeding reduced *Ffar2* and *Ffar3* expressions in DUE and ILE. However, quercetin supplementation restored *Ffar2* and *Ffar3* expressions to levels higher than those of LFD-fed mice. To examine whether quercetin-regulated intestinal integrity is linked to serotonin, a neurotransmitter hormone, serotonin transporter (*Sert*) and 5-hydroxytryptamine receptor 7 (*5-Htr7*) were assessed. In LFD-fed mice, quercetin upregulated *Sert* expression across all intestinal segments, but increased *5-Htr7* only in the JEJU (Figure 3I,J). HFD feeding reduced the expression of both *Sert* and *5-Htr7*. These reductions were prevented or even further enhanced by quercetin supplementation.

### 3.4. Effect of Quercetin on Signaling Pathways Involved in Intestinal Inflammation, Integrity, and Oxidative Stress

**Figure 4:**
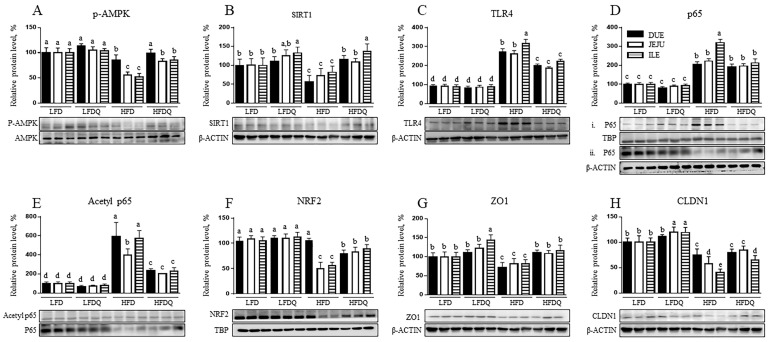
Effect of quercetin on proteins related to tight junction and inflammation in the intestine. (**A**) P-AMPK, (**B**) SIRT1, (**C**) TLR4, (**D**) NF-κB p65, (i) the nucleus and (ii) cytoplasm, (**E**) NF-κB p65 acetylation, (**F**) NRF2, (**G**) ZO1, and (**H**) CLDN1 proteins were determined in the intestine of mice. The lower panel of each graph shows a representative Western blot image. The P-AMPK was normalized by AMPK. SIRT1, TLR4, ZO1, and CLDN1 were normalized by β-ACTIN. NRF2, nuclear and cytosolic p65 proteins were normalized by TBP and β-ACTIN, respectively. NF-κB p65 acetylation was normalized to the NF-κB p65. Data are presented as mean ± SD (*n* = 9/group). Groups not sharing a common letter are significantly different according to Tukey’s post hoc test (*p* < 0.05).

To determine the mechanism by which quercetin alleviates inflammation and enhances tight junctions in the intestine of HFD-induced obese mice, proteins involved in the AMPK and NF-κB signaling pathways were examined (Figure 4A–E). As shown in Figure 4A, quercetin supplementation did not alter AMPK activity in LFD-fed mice. HFD feeding reduced AMPK phosphorylation by 18%, 43%, and 45% in the DUE, JEJU, and ILE, respectively, compared with LFD-fed mice. However, quercetin supplementation restored AMPK phosphorylation in HFD-fed mice to levels comparable to those of LFD-fed mice (Figure 4A). Quercetin supplementation increased SIRT1 protein levels in the ILE of LFDQ-fed mice but not in the DUE and JEJU. SIRT1 protein levels were significantly reduced in all intestinal segments of HFD-fed mice but were restored by quercetin (Figure 4B). HFD feeding significantly increased TLR4 protein levels by 172%, 163%, and 220% in the DUE, JEJU, and ILE, respectively, compared to LFD-fed mice. These increases were prevented by approximately 50% (*p* < 0.0002) (Figure 4C). HFD feeding increased nuclear p65 translocation by 104%, 123%, and 226% in the DUE, JEJU, and ILE, respectively. However, nuclear p65 translocation in ILE was only reduced by quercetin (Figure 4D). HFD also elevated p65 acetylation in all segments, whereas acetylation on p65 was reduced by quercetin (Figure 4E). HFD-fed mice showed decreased NRF2 protein levels by approximately 50% in the JEJU and ILE compared with LFD-fed mice. However, these reductions were alleviated in HFDQ-fed mice (Figure 4F). As shown in Figure 4G, LFDQ-fed mice showed a 43% increase in ILE ZO1 protein levels. HFD feeding reduced ZO1 protein, but this reduction was not observed in the intestine of HFDQ-fed mice. In the JEJU and ILE of LFD-fed mice, quercetin supplementation elevated CLDN1 protein levels. HFD feeding reduced CLDN1 protein by 24%, 43%, and 59% in the DUE, JEJU, and ILE, respectively, with the most significant reduction observed in ILE. These reductions were mitigated in both the JEJU and ILE of HFDQ-fed mice (Figure 4H).

### 3.5. Effect of Quercetin on miRNA Related to Tight Junction and Inflammation in the Intestine

**Figure 5:**
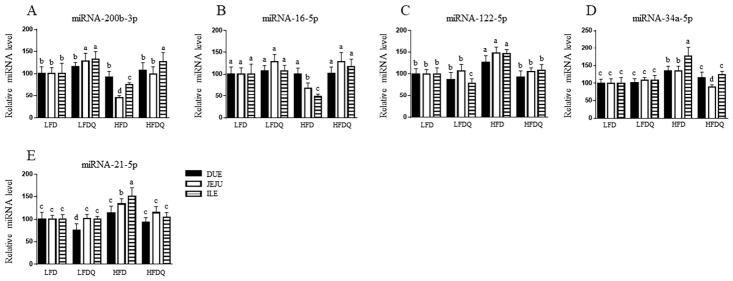
Effect of quercetin on miRNA related to tight junction and inflammation in the intestine. (**A**) miRNA-200b-3p, (**B**) miRNA-16-5p, (**C**) miRNA-122-5p, (**D**) miRNA-34a-5p, and (**E**) miRNA-21-5p. Data are presented as mean ± SD (*n* = 9/group). Groups not sharing a common letter are significantly different according to Tukey’s post hoc test (*p* < 0.05).

To determine whether the quercetin-mediated anti-inflammatory effect is associated with the regulation of miRNA, miRNAs involved in genes related to tight junction and inflammation were examined. In LFD-fed mice, quercetin supplementation increased miRNA-200b-3p expression in the JEJU and ILE by 28% and 33%, respectively (Figure 5A). HFD feeding reduced miRNA-200b-3p expression by 55% and 24% in the JEJU and ILE compared with LFD-fed mice. These reductions were not observed in HFDQ-fed mice (Figure 5A). As shown in Figure 5B, miRNA-16-5p expression decreased by 31% and 52% in the JEJU and ILE of HFD-fed mice. These reductions were prevented by quercetin supplementation. HFD feeding increased the expression of miRNA-122-5p, miRNA-34a-5p, and miRNA-21-5p in the intestine (Figure 5C–E). However, HFDQ-fed mice showed the same expression levels as LFD-fed mice.

## 4. Discussion

The intestinal tract functions as a primary interface between the external environment and the host, balancing nutrient absorption and immune defense. The ILE is recognized as the most immunoreactive segment due to its greater microbial exposure and the presence of Peyer’s patches [11], which may explain the more severe HFD-induced inflammation observed in ILE than in the DUE and JEJU in the present study. Correspondingly, in the present study, quercetin exerted its strongest anti-inflammatory effects in the ILE, although quercetin suppressed *Cox2*, *Ilb*, *Il6*, *Tnfa*, *Inos*, and *Mcp1* genes by reducing HFD-induced TLR4 and NF-κB activity across all intestinal segments. Consistent with our findings, LPS-challenged laying hens supplemented with quercetin (0.4 mg/kg) for 12 weeks exhibited reduced intestinal mucosal injury and necrosis, accompanied by decreased inflammation, as indicated by attenuated expression of *Tlr4* and *Il1b* in the JEJU and ILE, and increased expression of the anti-inflammatory cytokine *IL4* [5]. HFD-induced gut dysbiosis has been associated with increased luminal LPS production and activation of TLR4-mediated inflammatory signaling [12]. Consistent with this concept, quercetin supplementation reduced plasma LPS, LBP, I-FABP, and serum CD14 concentrations and intestinal TLR4 expression in the present study, suggesting attenuation of endotoxin translocation and TLR4-associated intestinal inflammation. These findings are in agreement with previous reports demonstrating that quercetin modulated gut microbial composition, including reductions in Proteobacteria abundance and the *Firmicutes* to *Bacteroidetes* ratio in HFD-fed mice [8,13], which may contribute to lower intestinal LPS exposure. Interestingly, quercetin restored miRNA-16-5p levels suppressed by HFD feeding in the present study, suggesting that its anti-inflammatory effects may be mediated, at least in part, by miR-16-dependent inhibition of TLR4/NF-κB signaling through post-transcriptional suppression of TLR4 and nuclear factor kappa B kinase subunit beta (IKBKB) expression [14,15,16]. In addition, phenolic compounds, chicoric acid, chlorogenic acid, 4′-*O*-demethylbroussonin A, and quercetin from dandelion (*Taraxacum officinale*) disrupted the formation of the TLR4-myeloid differentiation protein-2 (MD-2) complex either by binding hydrophobic core of TLR4’s leucine-rich repeats or by occupying hydrophobic pocket of MD2 where LPS normally binds [17], suggesting that quercetin may attenuate TLR4 signaling through multiple mechanisms. Although quercetin almost completely prevented the expression of proinflammatory genes in the present study, TLR4 expression was only partially reduced, implying the involvement of additional regulatory pathways. In the present study, quercetin also restored AMPK activity and SIRT1 expression which were diminished by HFD feeding. Given the role of AMPK, a central energy-sensing kinase that regulates metabolic homeostasis, oxidative stress responses, and cell survival, and its downstream effector, the nicotinamide adenine dinucleotide (NAD^+^)-dependent deacetylase, SIRT1, in suppressing NF-κB signaling by deacetylating the p65 subunit of NF-κB at lysine 310 [18], the AMPK-SIRT1 axis may provide an additional mechanism by which quercetin attenuates intestinal inflammation.

Because MPO activity is a marker of neutrophil-associated oxidative stress and intestinal inflammation [19], reduced intestinal and plasma MPO activities observed in HFD-fed mice suggest that quercetin attenuated inflammation-driven oxidative injury. This was accompanied by restoration of HFD-suppressed Nrf2, a key transcription factor that maintains redox balance and its downstream endogenous antioxidant enzymes including SOD, CAT, and GPX across intestinal segments. Notably, the expression of antioxidant genes was also elevated in LFDQ-fed mice, indicating that quercetin may enhance intrinsic redox defenses even in the absence of metabolic stress. The restoration of AMPK activity and SIRT1 expression by quercetin may contribute to enhanced Nrf2 signaling, as activation of the AMPK–SIRT1 axis promotes Nrf2 nuclear translocation and induces antioxidant gene expression [20,21]. Consistent with our findings, quercetin attenuated LPS-induced intestinal injury in the JEJU of broiler chickens and laying hens by reducing ROS, malondialdehyde, and 8-hydroxy-2′-deoxyguanosine, while enhancing activities of SOD and GPX as well as the expression of HO1 and NAD(P)H: quinone oxidoreductase 1 through the activation of the Nrf2 pathway via restoration of LPS-suppressed phosphorylation of c-Jun N-terminal kinase, extracellular signal-regulated kinase, and p38 mitogen-activate protein kinase [5,6]. Similarly, quercetin alleviated intestinal oxidative stress, inflammation, and barrier dysfunction in experimental models of colitis and cells through activation of Nrf2-mediated antioxidant defense, modulation of gut microbiota composition, and preservation of TJ integrity [22,23,24]. Taken together, our results suggest that quercetin mitigates intestinal oxidative injury at least in part, through activation of the AMPK–SIRT1–Nrf2 signaling axis.

AMPK activation also regulates barrier integrity by promoting TJ assembly, stabilizing the actin cytoskeleton, and enhancing epithelial differentiation [5]. Quercetin improved intestinal integrity in HFD-induced obese mice, and LPS (1 mg/kg) intraperitoneally injected laying hens [5,25]. Consistent with our previous findings in LPS-induced Caco-2/HT-29 cells [7], quercetin decreased HFD-induced intestinal permeability and increased the expression of TJ proteins, Muc2, and Muc6, accompanied by AMPK activation in the present study. Notably, TJ proteins were also increased in LFDQ-fed mice, indicating that quercetin promotes TJs under normal physiological conditions. Maintaining intestinal barrier integrity has been implicated with the involvement of miRNAs. miRNA-200b promotes intestinal barrier integrity by targeting myosin light chain kinase (MLCK) and stabilizing membrane-associated ZO1 and CLDN1, whereas miRNA-21 promotes epithelial permeability through downregulation of TJs, including OCLN, CLDNs, and ZO1. Similarly, miRNA-34 family members and miRNA-122 are associated with barrier dysfunction by modulating TJ-associated proteins and disrupting TJ assembly during intestinal inflammation [26,27,28,29,30]. In the present study, quercetin modulated the expression of barrier-related miRNAs by reducing the expression of HFD-induced miRNA-21-5p, miRNA-34a-5p, and miRNA-122-5p and restoring the expression of HFD-suppressed miRNA-200b-3p, suggesting that quercetin may preserve intestinal barrier integrity, at least in part, through coordinated post-translational regulation of TJ stability and assembly. Although quercetin restored TJ gene and protein expression to normal or higher levels in HFD-fed mice, intestinal permeability was reduced by only 17%, indicating that increased expression alone is insufficient to fully restore barrier function. TJ function is also influenced by claudin composition, membrane localization, and post-translational regulation, all of which are critical for proper junction assembly and barrier sealing [31,32,33,34]. Therefore, while quercetin appears to regulate TJ proteins at the transcriptional and post-transcriptional levels, its effects on TJ assembly, localization, and post-translational regulation remain to be elucidated.

Based on our previous observation that quercetin modulated the gut microbiota and increased SCFA production in the same model [8], the present findings suggest that these microbial changes may contribute to improved intestinal barrier function and reduced inflammation. SCFAs activate FFAR2 and FFAR3 (GPR43 and GPR41) on intestinal epithelial, immune, and enteroendocrine cells to stimulate the secretion of satiety hormones GLP1 and PYY, which suppress inflammatory signaling and enhance epithelial barrier function by increasing TJ protein and MUC2 production through AMPK activation [35,36,37,38]. Thus, the restored expression of *Ffar2*, and *Ffar3* and increased circulating GLP1 levels in HFDQ-fed mice may reflect enhanced SCFA-mediated enteroendocrine signaling that contributes to the attenuation of intestinal inflammation and preservation of barrier integrity. Microbiota-derived SCFAs also regulate intestinal serotonergic homeostasis through modulation of SERT-mediated serotonin reuptake and 5-HTR signaling, which supports epithelial repair, anti-inflammatory responses, and mucosal barrier function [39,40]. Accordingly, the restoration of *Sert* and *5-Htr7* expressions in HFDQ-fed mice suggests that quercetin-induced microbial alterations may enhance serotonergic signaling to preserve intestinal homeostasis.

## 5. Conclusions

Quercetin improves gut barrier integrity in HFD-fed mice by enhancing tight junction expression, reducing inflammation and oxidative stress in the coordinated post-transcriptional mechanisms via AMPK-mediated NF-κB and NRF2 signaling pathways and modulating gut hormone levels, thus lowering circulating endotoxemia markers. Importantly, quercetin also enhances tight junction and antioxidant enzyme expression under normal conditions. This suggests quercetin has a potential role for both the protection and maintenance of intestinal homeostasis.

## Data Availability

The data are available from the corresponding author upon reasonable request.

## References

[1] Turner J.R. (2009). Intestinal mucosal barrier function in health and disease. Nat. Rev. Immunol..

[2] Wells J.M., Brummer R.J., Derrien M., MacDonald T.T., Troost F., Cani P.D., Theodorou V., Dekker J., Meheust A., de Vos W.M. (2017). Homeostasis of the gut barrier and potential biomarkers. Am. J. Physiol. Gastrointest. Liver Physiol..

[3] Buckley A., Turner J.R. (2018). Cell Biology of Tight Junction Barrier Regulation and Mucosal Disease. Cold Spring Harb. Perspect. Biol..

[4] Jamieson P.E., Carbonero F., Stevens J.F. (2023). Dietary (poly) phenols mitigate inflammatory bowel disease: Therapeutic targets, mechanisms of action, and clinical observations. Curr. Res. Food Sci..

[5] Feng J., Li Z., Ma H., Yue Y., Hao K., Li J., Xiang Y., Min Y. (2023). Quercetin alleviates intestinal inflammation and improves intestinal functions via modulating gut microbiota composition in LPS-challenged laying hens. Poult. Sci..

[6] Sun L., Xu G., Dong Y., Li M., Yang L., Lu W. (2020). Quercetin protects against lipopolysaccharide-induced intestinal oxidative stress in broiler chickens through activation of Nrf2 pathway. Molecules.

[7] Balogun O., Brownmiller C.R., Lee S.-O., Kang H.W. (2024). Onion Peel Extract Prevents Intestinal Inflammation via AMK-Activated Protein Kinase Activation in Caco-2/HT-29 Cells. Nutrients.

[8] Pei Y., Otieno D., Gu I., Lee S.O., Parks J.S., Schimmel K., Kang H.W. (2021). Effect of quercetin on nonshivering thermogenesis of brown adipose tissue in high-fat diet-induced obese mice. J. Nutr. Biochem..

[9] Rubio C., Puerto M., García-Rodríquez J.J., Lu V.B., García-Martínez I., Alén R., Sanmartín-Salinas P., Toledo-Lobo M.V., Saiz J., Ruperez J. (2020). Impact of global PTP1B deficiency on the gut barrier permeability during NASH in mice. Mol. Metab..

[10] Pei Y., Parks J.S., Kang H.W. (2021). Quercetin alleviates high-fat diet-induced inflammation in brown adipose tissue. J. Funct. Foods.

[11] Mach N., Berri M., Esquerre D., Chevaleyre C., Lemonnier G., Billon Y., Lepage P., Oswald I.P., Dore J., Rogel-Gaillard C. (2014). Extensive expression differences along porcine small intestine evidenced by transcriptome sequencing. PLoS ONE.

[12] Wei J., Zhang Y., Li H., Wang F., Yao S. (2023). Toll-like receptor 4: A potential therapeutic target for multiple human diseases. Biomed. Pharmacother..

[13] Porras D., Nistal E., Martínez-Flórez S., Pisonero-Vaquero S., Olcoz J.L., Jover R., González-Gallego J., García-Mediavilla M.V., Sánchez-Campos S. (2017). Protective effect of quercetin on high-fat diet-induced non-alcoholic fatty liver disease in mice is mediated by modulating intestinal microbiota imbalance and related gut-liver axis activation. Free Radic. Biol. Med..

[14] Li X., Chu Q., Wang H. (2021). MicroRNA-16 regulates lipopolysaccharide-induced inflammatory factor expression by targeting TLR4 in normal human bronchial epithelial cells. Exp. Ther. Med..

[15] Xi M., Zhao P., Li F., Bao H., Ding S., Ji L., Yan J. (2022). MicroRNA-16 inhibits the TLR4/NF-κB pathway and maintains tight junction integrity in irritable bowel syndrome with diarrhea. J. Biol. Chem..

[16] Tang X., Jin L., Cao P., Cao K., Huang C., Luo Y., Ma J., Shen S., Tan M., Li X. (2016). MicroRNA-16 sensitizes breast cancer cells to paclitaxel through suppression of IKBKB expression. Oncotarget.

[17] Zou H., Ben T., Wu P., Waterhouse G.I., Chen Y. (2023). Effective anti-inflammatory phenolic compounds from dandelion: Identification and mechanistic insights using UHPLC-ESI-MS/MS, fluorescence quenching and anisotropy, molecular docking and dynamics simulation. Food Sci. Hum. Wellness.

[18] Xu Y., Bai L., Yang X., Huang J., Wang J., Wu X., Shi J. (2024). Recent advances in anti-inflammation via AMPK activation. Heliyon.

[19] Singh S., Anshita D., Ravichandiran V. (2021). MCP-1: Function, regulation, and involvement in disease. Int. Immunopharmacol..

[20] Petsouki E., Cabrera S.N.S., Heiss E.H. (2022). AMPK and NRF2: Interactive players in the same team for cellular homeostasis?. Free Radic. Biol. Med..

[21] Mo C., Wang L., Zhang J., Numazawa S., Tang H., Tang X., Han X., Li J., Yang M., Wang Z. (2014). The crosstalk between Nrf2 and AMPK signal pathways is important for the anti-inflammatory effect of berberine in LPS-stimulated macrophages and endotoxin-shocked mice. Antioxid. Redox Signal..

[22] Dong Y., Hou Q., Lei J., Wolf P.G., Ayansola H., Zhang B. (2020). Quercetin alleviates intestinal oxidative damage induced by H2O2 via modulation of GSH: In vitro screening and in vivo evaluation in a colitis model of mice. ACS Omega.

[23] Liu W., Zhou Y., Qin Y., Yu L., Li R., Chen Y., Xu Y. (2020). Quercetin intervention alleviates offspring’s oxidative stress, inflammation, and tight junction damage in the colon induced by maternal fine particulate matter (PM2. 5) exposure through the reduction of Bacteroides. Nutrients.

[24] Lv Y., Peng J., Ma X., Liang Z., Salekdeh G.H., Ke Q., Shen W., Yan Z., Li H., Wang S. (2024). Dietary quercetin protects against dextran sodium sulfate (DSS)-induced colitis mice by restoring intestinal barrier, Reducting oxidative stress and inflammation via modulating gut microbiota. Res. Sq..

[25] Su L., Zeng Y., Li G., Chen J., Chen X. (2022). Quercetin improves high-fat diet-induced obesity by modulating gut microbiota and metabolites in C57BL/6J mice. Phytother. Res..

[26] Shen Y., Zhou M., Yan J., Gong Z., Xiao Y., Zhang C., Du P., Chen Y. (2017). miR-200b inhibits TNF-α-induced IL-8 secretion and tight junction disruption of intestinal epithelial cells in vitro. Am. J. Physiol. Gastrointest. Liver Physiol..

[27] He W.-Q., Wang J., Sheng J.-Y., Zha J.-M., Graham W.V., Turner J.R. (2020). Contributions of myosin light chain kinase to regulation of epithelial paracellular permeability and mucosal homeostasis. Int. J. Mol. Sci..

[28] Zhang L., Shen J., Cheng J., Fan X. (2015). MicroRNA-21 regulates intestinal epithelial tight junction permeability. Cell Biochem. Funct..

[29] Chen T., Xue H., Lin R., Huang Z. (2017). MiR-34c and PlncRNA1 mediated the function of intestinal epithelial barrier by regulating tight junction proteins in inflammatory bowel disease. Biochem. Biophys. Res. Commun..

[30] Ye D., Guo S., Al R., Ma T.Y. (2011). MicroRNA Regulation of Intestinal Epithelial Tight Junction Permeability. Gastroenterology.

[31] Overgaard C.E., Daugherty B.L., Mitchell L.A., Koval M. (2011). Claudins: Control of barrier function and regulation in response to oxidant stress. Antioxid. Redox Signal..

[32] Poritz L.S., Harris L.R., Kelly A.A., Koltun W.A. (2011). Increase in the tight junction protein claudin-1 in intestinal inflammation. Dig. Dis. Sci..

[33] Balda M.S., Whitney J.A., Flores C., González S., Cereijido M., Matter K. (1996). Functional dissociation of paracellular permeability and transepithelial electrical resistance and disruption of the apical-basolateral intramembrane diffusion barrier by expression of a mutant tight junction membrane protein. J. Cell Biol..

[34] Elias B.C., Suzuki T., Seth A., Giorgianni F., Kale G., Shen L., Turner J.R., Naren A., Desiderio D.M., Rao R. (2009). Phosphorylation of Tyr-398 and Tyr-402 in occludin prevents its interaction with ZO-1 and destabilizes its assembly at the tight junctions. J. Biol. Chem..

[35] Li Y., Huang Y., Liang H., Wang W., Li B., Liu T., Huang Y., Zhang Z., Qin Y., Zhou X. (2023). The roles and applications of short-chain fatty acids derived from microbial fermentation of dietary fibers in human cancer. Front. Nutr..

[36] Wang W., Zhang C., Zhang H., Li L., Fan T., Jin Z. (2023). The alleviating effect and mechanism of GLP-1 on ulcerative colitis. Aging.

[37] Gao J., Mang Q., Sun Y., Xu G. (2025). Short-chain fatty acids (SCFAs) modulate the hepatic glucose and lipid metabolism of coilia nasus via the FFAR/AMPK signaling pathway In vitro. Int. J. Mol. Sci..

[38] Burger-van Paassen N., Vincent A., Puiman P.J., van Der Sluis M., Bouma J., Boehm G., Van Goudoever J.B., Van Seuningen I., Renes I.B. (2009). The regulation of intestinal mucin MUC2 expression by short-chain fatty acids: Implications for epithelial protection. Biochem. J..

[39] Buey B., Forcén A., Grasa L., Layunta E., Mesonero J.E., Latorre E. (2023). Gut microbiota-derived short-chain fatty acids: Novel regulators of intestinal serotonin transporter. Life.

[40] Wang A., Li P., Ma F., Li X., Mu G., Tuo Y. (2023). Mixed Lactiplantibacillus plantarum strains alleviated DSS-induced intestinal inflammation of Balb/c mice via the 5-HT/5-HT7R/NF-κB signaling pathway. J. Funct. Foods.

